# Regulatory T Cells and Pro-inflammatory Responses Predominate in Children with Tuberculosis

**DOI:** 10.3389/fimmu.2017.00448

**Published:** 2017-04-25

**Authors:** Elizabeth Whittaker, Mark Nicol, Heather J. Zar, Beate Kampmann

**Affiliations:** ^1^Academic Department of Paediatrics, Imperial College London, London, UK; ^2^UCT Faculty of Health Sciences, Division of Medical Microbiology, Department of Clinical Laboratory Sciences, Institute of Infectious Disease and Molecular Medicine, Cape Town, South Africa; ^3^MRC Unit of Child and Adolescent Health, University of Cape Town, Cape Town, South Africa; ^4^Department of Paediatrics and Child Health, Red Cross War Memorial Children’s Hospital, Cape Town, South Africa; ^5^Vaccines and Immunity Theme, MRC Unit The Gambia, Fajara, Gambia

**Keywords:** tuberculosis, extrapulmonary, pediatric, mycobacterial immunity, regulatory T cells

## Abstract

**Background:**

Following infection with *Mycobacterium tuberculosis* (M.tb), children are more susceptible to develop disease particularly extrapulmonary disease than adults. The exact mechanisms required for containment of M.tb are not known, but would be important to identify correlates of protection.

**Objective:**

To comprehensively analyze key immune responses to mycobacteria between HIV-negative children with extrapulmonary TB (EPTB) compared to children with pulmonary TB (PTB) or healthy controls.

**Methods:**

Whole blood was stimulated *in vitro* with mycobacteria for 24 h or 6 days to induce effector and memory responses. CD4, CD8, γδ, regulatory T cells, and their related cytokines were measured. Samples of children with tuberculosis (TB) disease were analyzed both at time of diagnosis and at the end of TB treatment to determine if any differences were due to TB disease or an underlying host phenotype.

**Results:**

Seventy-six children with TB disease (48 with PTB and 28 with EPTB) and 83 healthy controls were recruited to the study. The frequency of CD4^+^CD25^+^CD39^+^FOXP3^+^ regulatory T cells and secreted IL10 were significantly higher in children with TB compared to healthy controls. IFNγ-, IL17-, and IL22-producing γδ T cells, IL22-producing CD4^+^ T cells and secreted pro-inflammatory cytokines (IFNγ, IL1β, and TNFα) were significantly lower in children with TB disease compared to healthy controls. IFNγ-producing CD4^+^ T cells and Ki67^+^-proliferating CD4^+^ T cells, however, were present in equal numbers in both groups. Following treatment, these immune parameters recovered to “healthy” levels or greater in children with PTB, but not those with extrapulmonary TB.

**Conclusion:**

In children with TB disease, a predominantly immune regulatory state is present. These immune findings do not distinguish between children with PTB and EPTB at the time of diagnosis. Following treatment, these inflammatory responses recover in PTB, suggesting that the effect is disease specific rather than due to an underlying host defect.

## Introduction

Of the estimated 10.4 million new cases of tuberculosis (TB) annually, at least 1million (10%) occurred in children (1). The BCG vaccine confers partial protection against disseminated TB in young children but has variable efficacy against pulmonary TB (PTB), and better vaccines are urgently required. Following infection, children have a higher risk not only of progression to disease but also of dissemination or extrapulmonary TB (EPTB) and death, and this risk decreases with increasing age (2, 3). Characterizing the mycobacterial-specific immune responses in children affected by either PTB or EPTB may provide insight into the mechanisms of immune containment, which are essential in the search for correlates of protection, so urgently required for a better vaccine against TB.

T cell immunity is well recognized as essential for protection against TB infection and disease, and CD4^+^ T cell depletion, such as seen in HIV, is a contributing factor to TB susceptibility. Mycobacterial-specific CD4^+^ T cells primarily produce Th1 cytokines, which include IFNγ, IL-2, and TNFα. However, recent studies report a lack of correlation between immune protection imparted by BCG and IFNγ, produced by CD4^+^ T cells (4, 5). Hence, these data support the notion that although CD4^+^ T cells and IFNγ are important components of an effective anti-mycobacterial immune response, they do not fully explain observed differences in host susceptibility to TB. Other cell types, such as γδ, Th17, and regulatory T cells are also considered important, but these T cell populations have not been studied in children with PTB or EPTB (6, 7).

Animal models have established that the presence of an excessive number of γδ T cells producing IL17 lead to severe disseminated disease (8, 9). It is likely in humans that the balance between mycobacterial antigen-specific IL17 and IFNγ-producing T cells is also of importance in protection against TB disease and in particular, EPTB. This has not been examined in children but would be compatible with their known higher susceptibility to progress to pulmonary and disseminated disease, given the relatively impaired production of IFNγ in young age (10).

Increased numbers of regulatory T cells have been found in adults with TB infection and disease in comparison to healthy controls, particularly in patients with EPTB, a finding that persists even in patients with “cured” EPTB (11–13). Regulatory T cells expressing FOXP3 correlate well with regulatory activity; however, the FOXP3 marker is also present in activated T cells in the presence of IL2. Recently, CD39 was found to be expressed on a subpopulation of regulatory T cells. CD39 is an ectonucleotidase that cleaves ATP in a rate-limiting step to form AMP, which can then be cleaved by CD73 to form adenosine. Extracellular ATP has multiple pro-inflammatory effects, and its removal may, therefore, have a net anti-inflammatory influence. CD4^+^CD25^+^CD39^+^FoxP3^+^ cells are found to suppress IL17 production and are increased in TB patients after antigen-specific stimulation. Depletion of these cells resulted in increased antigen-specific IFNγ CD4 T cell responses (14). We chose to measure CD4^+^CD25^+^CD39^+^FOXP3^+^ T cells based on the most recent literature, suggesting that these markers most accurately identify functionally suppressive regulatory T cells in humans, influence mycobacterial specific responses in patients with TB disease and play a role in influencing the immune response to novel TB vaccines (15, 16). To date, there are no studies that have examined the role of regulatory T cells in different clinical manifestations of pediatric TB.

Based on these interesting data, we hypothesized that the mycobacterial-specific immune response in children with TB, in particular EPTB, would have an increased frequency of both IL17-producing γδ T cells and regulatory T cells in comparison with healthy controls. We aimed to characterize these T cell populations in a group of HIV-negative children with PTB and EPTB compared to healthy children without any evidence of sensitization to *Mycobacterium tuberculosis* (M.tb). The aim of this study was to compare immune responses to mycobacteria in children with PTB, EPTB, and healthy controls.

Our data show that regulatory T cells are increased in children with TB disease compared to healthy controls, but do not discriminate between pulmonary and extrapulmonary TB disease. This is associated with suppression of pro-inflammatory cytokines, which corresponds with recent gene expression studies (17–20). Furthermore, as these immune responses were also examined following treatment, we can now confirm they are TB-disease specific, rather than a product of young age or host differences.

## Materials and Methods

### Participant Enrollment and Classification

The study was conducted at Red Cross Children’s Hospital (RCH) in Cape Town, South Africa. Children between birth and 16 years were enrolled to three different groups: pulmonary TB (PTB) disease, extrapulmonary TB (EPTB) disease, and healthy, non-M.tb sensitized children. Children with TB disease presented to RCH with symptoms consistent with active TB and the following clinical details were evaluated: presenting symptoms, history of TB contact, CXR/radiological findings, microscopy, and culture for M.tb, and clinical examination findings. Children with culture-confirmed or GeneXpert-positive disease were classified as “confirmed” TB. Children who fitted the clinical criteria, but did not have microbiological confirmation, were treated for TB disease, and responded to treatment were classified as “unconfirmed” TB. TB disease extending beyond the pleural cavity was classified as EPTB. Children were excluded if they had received treatment for TB lasting longer than 72 h, they did not live in Cape Town, they were unable to attend follow-up visits, or informed consent was not given.

Healthy controls were recruited from well children who presented to RCH for elective surgical interventions. An in-house interferon gamma release assay (IGRA) was used to determine M.tb sensitization status; children with a positive IGRA were further evaluated for active TB but not included in this analysis. Children with a negative IGRA were classified as healthy controls. Exclusion criteria for healthy controls were known contact with TB; prior treatment for TB, history of recurrent infections, or hospital admissions; persistent cough for longer than 4 weeks; intercurrent febrile illness; and failure to thrive or known immunodeficiency. All children had received BCG vaccination at birth, and only HIV-negative children were included in the study.

### Blood Collection, Stimulation, and Cryopreservation

Blood samples from children with TB disease were taken within 48 h of enrollment and repeated after completion of 6 months treatment.

Heparinized blood was incubated within 4 h with BCG (SSI strain, 5 × 10^5^cfu/ml), as previously described (21). Medium served as negative control; staphylococcal enterotoxin B (SEB, 10 µg/ml final concentration; Sigma, UK) was used as positive control. Brefeldin-A was added for the last 5 h of the 20-h incubation. Cells were then harvested, fixed, and cryopreserved.

It was not possible to evaluate FOXP3 expression directly *ex vivo*. FOXP3^+^ regulatory T cells were identified in unstimulated whole blood which was incubated for 20 h, including with Brefeldin for the final 5 h.

A further aliquot of whole blood (diluted 1:10 in RPMI 1640) was incubated with BCG (SSI, 2.5 × 10^6^ cfu/ml) for 6 days for a Ki67 lymphoproliferation assay as previously described (22). Medium and SEB (5 µg/ml final concentration) served as negative and positive controls, respectively. To assess intracellular cytokine production, 10 ng/ml phorbol 12-myristate 13-acetate (PMA, Sigma-Aldrich), 1.5 µg/ml ionomycin (Sigma-Aldrich), and 1.5 µg/ml Brefeldin A were added during the last 5 h of culture. Cells were then harvested, stained with a viability dye, fixed, and cryopreserved.

Prior to the addition of PMA/Ionomycin/Brefeldin, 500 µl of supernatant was removed and stored at −80°C for subsequent analysis by multiplex ELISA (Bio-plex Pro™ Human Th17 Cytokine Panel) to determine levels of secreted cytokines.

### Cell Staining and Flow Cytometric Analysis

Cryopreserved cells were thawed, washed, and permeabilized with Perm/Wash solution (BD Biosciences). Cells were then incubated at 4°C for 1 h with fluorescence-conjugated antibodies directed against surface antigens and intracellular cytokines. For detection of the transcription factor FOXP3, a nuclear permeabilization buffer (eBioscience, San Diego, CA, USA) was used in place of the Perm/Wash solution as per manufacturer’s protocol. The following fluorescence-conjugated antibodies were used: anti-CD3 PacBlue, anti-γδ TCR PE, anti-CD27 PECy7, anti-CD25 APC, anti-IFNγ Alexafluor 700, anti-IL17A Alexafluor 647, anti-Ki67FITC (BD biosciences, San Jose, CA, USA); anti-CD45RA QDot655, anti-CD8 QDot605, anti-CD4 QDot605 (Invitrogen, Eugene, OR, USA); and anti-CD39 FITC, anti-IL22 PerCP-Efluor710, and anti-FOXP3 PE (eBioscience, SanDiego, CA, USA).

The entire sample was acquired on a BD LSRFortessa Flow Cytometer using FACSDiva software (BD Biosciences, San Jose, CA, USA). Compensation was performed using compensation beads.

### Secreted Cytokine Measurement

Supernatants from whole blood stimulated for 6 days with BCG were thawed. Concentrations of IL-1β, IL-4, IL-6, IL-10, IL-17, IL-22, IL-23, IFNγ, and TNFα were measured for all samples by Milliplex MAP Multiplex Immunoassay (based on Luminex MAP technology; Millipore) on a Bio-Rad Luminex 100 Bio-Plex Liquid Array Multiplexing System Fluorescent Reader, according to the manufacturer’s instruction. The following concentrations of the standards in 200 ml assay buffer were used: 10,000, 2,000, 400, 80, 16, and 3.2 pg/ml.

### Data Analysis

All flow cytometry data was analyzed using FlowJo v9.4.11 (TreeStar, Ashland, OR, USA). Combinations of antigen-specific cytokine-producing cells were determined by Boolean Gating in FlowJo.

SPSS (version 21) and GraphPad Prism (version 5.0a, 2008) were used for statistical analysis of multiparameter flow cytometry data. Negative control values for cytokine expression were subtracted from BCG-induced responses. An empiric cutoff value of 0.01% was defined as positive (23). Differences between groups were calculated using either Mann–Whitney or Kruskal–Wallis analysis of variance. For correlations, a Spearman coefficient for non-parametric data was calculated. All tests were two-tailed, and a value of *p* < 0.05 was considered significant.

## Results

### Clinical Characteristics of Patients with TB Disease and Healthy Children

One hundred fifty-nine children were recruited; 76 had TB disease with a broad clinical spectrum of which 42 (55%) were confirmed, 83 were healthy controls. The demographic and clinical characteristics are shown in Table 1. There was no significant difference in median age between children with PTB and those with EPTB; however, the healthy children were younger.

**Table 1:** **Demographic and clinical characteristics of study population**.

	Tuberculosis (TB) disease		Healthy controls *n* = 83
	Pulmonary *n* = 48	Extrapulmonary *n* = 28	
Median age in years (range)	3.75 (0.2–13)	5.2 (0.5–11.75)	2 (0.3–11.1)
Female gender (%)	20 (42)	13 (45)	30 (36)
TST in mm (range)	14 (0–34)	14 (0–30)	NA
Culture or GeneXpert confirmation (%)	23 (48)	19 (68)	NA
CNS disease (%)	NA	8 (29)	NA
Abdominal disease (%)	NA	7 (25)	NA
Pleural effusion (%)	NA	7 (25)	NA
Other (pericarditis, bone/joint, skin, genitourinary) (%)	NA	7 (25)	NA
MDR TB (%)	4 (8)	3 (11)	NA

*Some children had more than one clinical manifestation of extrapulmonary TB, hence % add up to greater than 100%*.

*TST, tuberculin skin test; CNS, central nervous system; MDR, multidrug-resistant tuberculosis*.

We first investigated CD4 T cell responses in children with confirmed and unconfirmed TB and did not find any significant differences. These two groups were subsequently combined to analyze differences between the TB cohort and healthy controls before proceeding to analyses according to disease phenotypes.

### Antigen-Specific CD4^+^, γδ^+^, and CD8^+^ T Cell Cytokine Production and Proliferation

**Figure 1:**
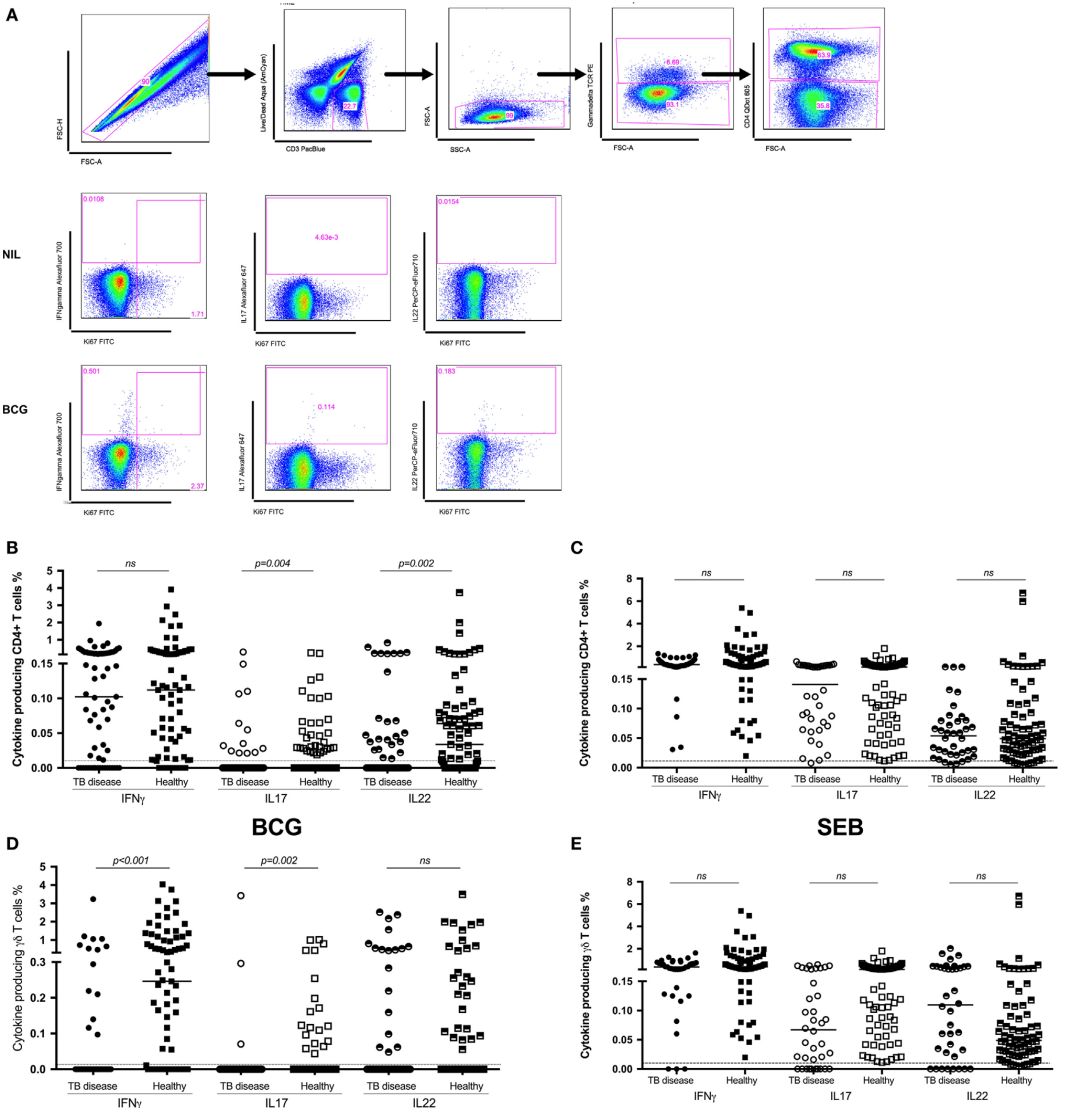
**Antigen-specific cytokine production in children with tuberculosis (TB) disease compared to healthy children**. **(A)** Gating strategy. Representative dot plots from a child healthy control are shown. From left to right, cell doublets were excluded with forward scatter area vs forward scatter height parameters. Next, dead cells as stained by a viability stain are gated out and live CD3 T cells gated in. Plotting FSC-A vs SSC-A allows confirmation of the lymphocyte population based on size and granularity. Plotting γδ T cell receptor vs FSC-A followed by CD4 vs FSC-A allows the immunophenotyping of these two different cell types. Representative dot plots of cytokine expression (IFNγ, IL17, and IL22 in CD4^+^ T cells) from unstimulated and BCG-stimulated conditions in a child with TB are shown. Gates were set using an unstimulated control. Frequencies of IFNγ, IL17, and IL22 producing CD4^+^ **(B,C)** and γδ^+^ **(D,E)** T cells following stimulation of whole blood with BCG **(B,D)** or SEB **(C,E)** for 20 h are shown. Responses in TB diseased (*n* = 76) and healthy children (*n* = 83) were compared. Horizontal bars represent median values. The dotted line represents a cutoff of 0.01 for a positive response. *p*-Values calculated by Mann–Whitney are displayed.

Following *in vitro* stimulation with BCG, production of IFNγ by CD4^+^ T cells was not significantly different between children with TB disease compared to healthy children [0.1% (IQR 0.0–0.24)% vs 0.11% (IQR 0.02–0.28); *p* = 0.5]. BCG-specific production of IL17 by CD4^+^ T cell was decreased in children with TB disease compared to healthy. However, the number of non-responders in both groups was very high and the percentage of cytokine producing cells very low, hence this should be interpreted with caution. BCG-specific CD4^+^ T cell production of IL22 was significantly suppressed in children with TB disease compared to healthy children [0% (IQR 0–0.04) vs 0.03% (IQR 0–0.08); *p* = 0.0017] (Figure 1B).

IFNγ production by γδ T cells was significantly lower in children with TB disease compared to healthy children [0% (IQR 0–0) vs 0.24% (IQR 0–1.18); *p* < 0.0001], possibly due to a high number of non-responders in the TB cohort. The pattern was the same for IL17 but not for IL22 (Figure 1D).

We also analyzed the CD4^+^ and γδ^+^ T cell responses induced by the positive control, SEB (Figures 1C,E), in order to exclude generalized non-responsiveness of T cell populations and found no significant differences between TB disease and healthy children for either cell type or cytokines with good responses to this non-specific antigen throughout.

Antigen-specific production of IFNγ, IL17, and IL22 by CD8^+^ T cells was lower in children with TB disease compared to healthy children, but there were very high numbers of non-responders in all groups (data not shown).

In order to determine potential differences in BCG-specific proliferative capacity and cytokine production between the groups, a 6-day proliferation assay employing Ki67 was also used. Heterogeneity of responses was considerable, but there were no significant differences between children with TB disease and healthy controls (data not shown).

### Regulatory T Cells Distinguish Children with TB Disease from Healthy Children

**Figure 2:**
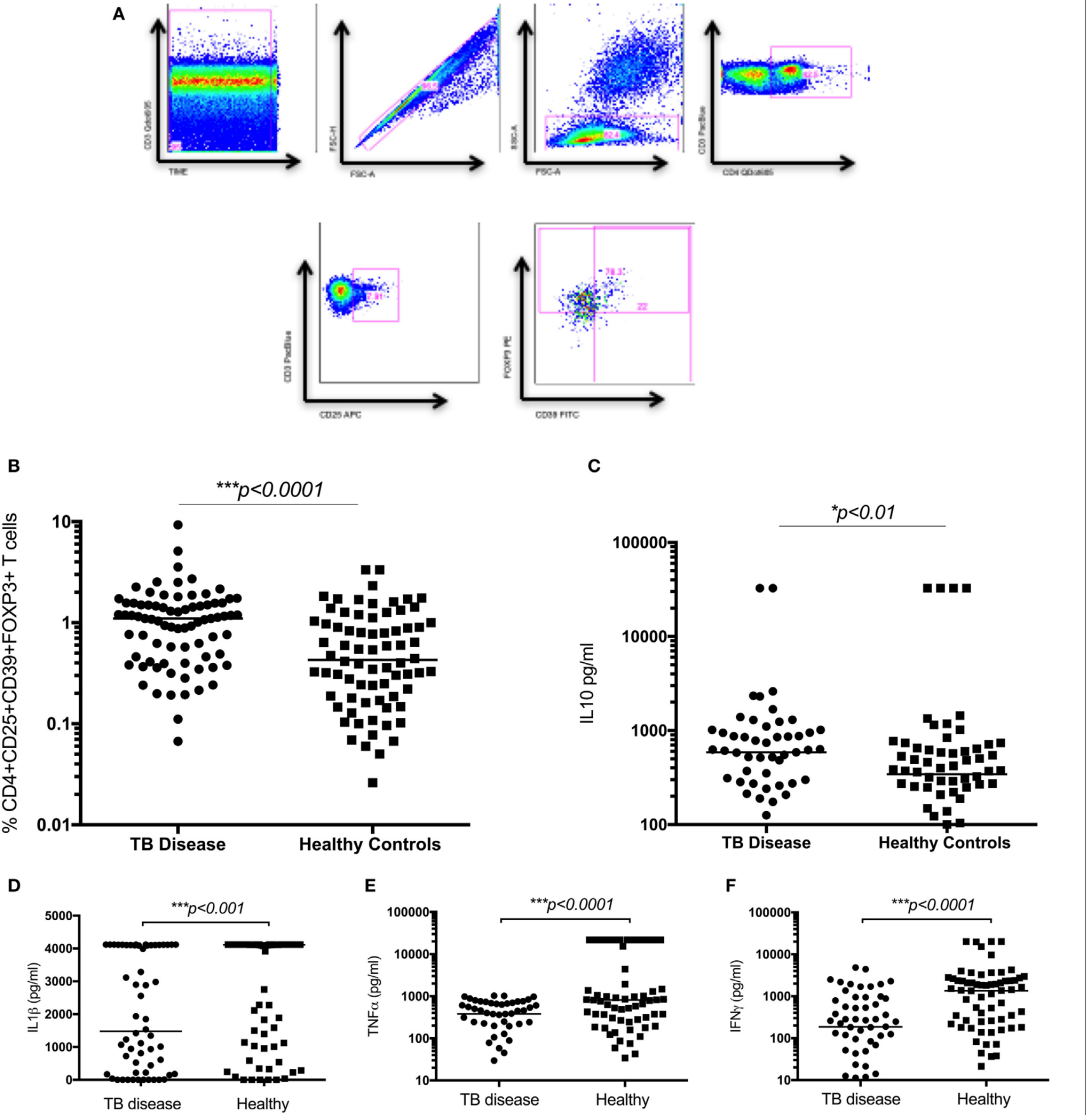
**Regulatory T cells and the related cytokine IL10 are significantly increased in children with tuberculosis (TB) disease compared to healthy controls**. **(A)** Gating strategy. From left to right, CD3 vs Times is shown to detect differences in flow. Cell doublets were excluded with forward scatter area vs forward scatter height parameters. Plotting FSC-A vs SSC-A allows identification of the lymphocyte population based on size and granularity. CD4^+^CD3^+^ T cells are identified, followed by CD4^+^CD25^+^ T cells. Using FMO gates, FOXP3 and CD39 gates are placed to identify the CD3^+^CD4^+^CD25^+^CD39^+^FOXP3^+^ population. Frequency of CD4^+^CD25^+^CD39^+^FOXP3^+^ regulatory T cells in unstimulated whole blood **(B)** and the secreted level of a key cytokines following 6 days of stimulation with BCG **(C–F)** are shown. The assays were performed for both groups: TB disease (*n* = 76) and healthy children (*n* = 83). Horizontal bars represent median values. *p*-Values calculated by a Mann–Whitney test are displayed.

The frequency of CD4^+^CD25^+^CD39^+^FOXP3^+^ T cells were significantly increased in children with TB disease compared to healthy controls [1.1% (IQR 0.46–1.56) vs 0.43% (IQR 0.18–1.01); *p* < 0.0001] (Figure 2B). Secreted IL10 was measured in supernatants to explore a possible functional correlate of this T cell population and was found to be significantly higher in children with TB disease compared to healthy controls [586 pg/ml (IQR 265–982) vs 343 pg/ml (IQR 118–625); *p* < 0.01] (Figure 2C). A range of BCG-induced pro-inflammatory cytokines were also measured with a number of pro-inflammatory cytokines significantly lower in children with TB disease compared to healthy controls (Figures 2D–F; Table 2).

**Table 2:** **Secreted cytokine production following BCG stimulation in healthy children vs those with TB disease**.

Cytokine	Healthy median (IQR) pg/ml	Active TB disease median (IQR) pg/ml	*p*-Value
IL1β	4,111 (1,056–4,118)	1,479 (206–4,107)	0.001
IL17F	1,283 (211–4,779)	240 (26–1,543)	<0.0001
IFNγ	1,357 (180–2,379)	186 (19–817)	<0.0001
TNFα	815 (270–21,848)	380 (73–671)	<0.0001
IL10	321 (101–615)	586 (265–982)	0.01
IL6	26,914 (7,390–27,065)	24,140 (9,673–28,354)	0.1
IL22	141 (60–337)	158 (51–339)	0.1
IL23	74 (17–475)	54 (0–242)	0.09
IL4	6.4 (2–19.3)	5.9 (1–42.6)	0.6

*Median secreted cytokine levels (picograms per milliliter) with interquartile range for each group is shown. A Mann–Whitney test for all comparisons was performed, *p*-values are shown*.

### T Cell Assays Do Not Distinguish between Children with Pulmonary and Extrapulmonary TB Disease at the Time of Diagnosis

**Figure 3:**
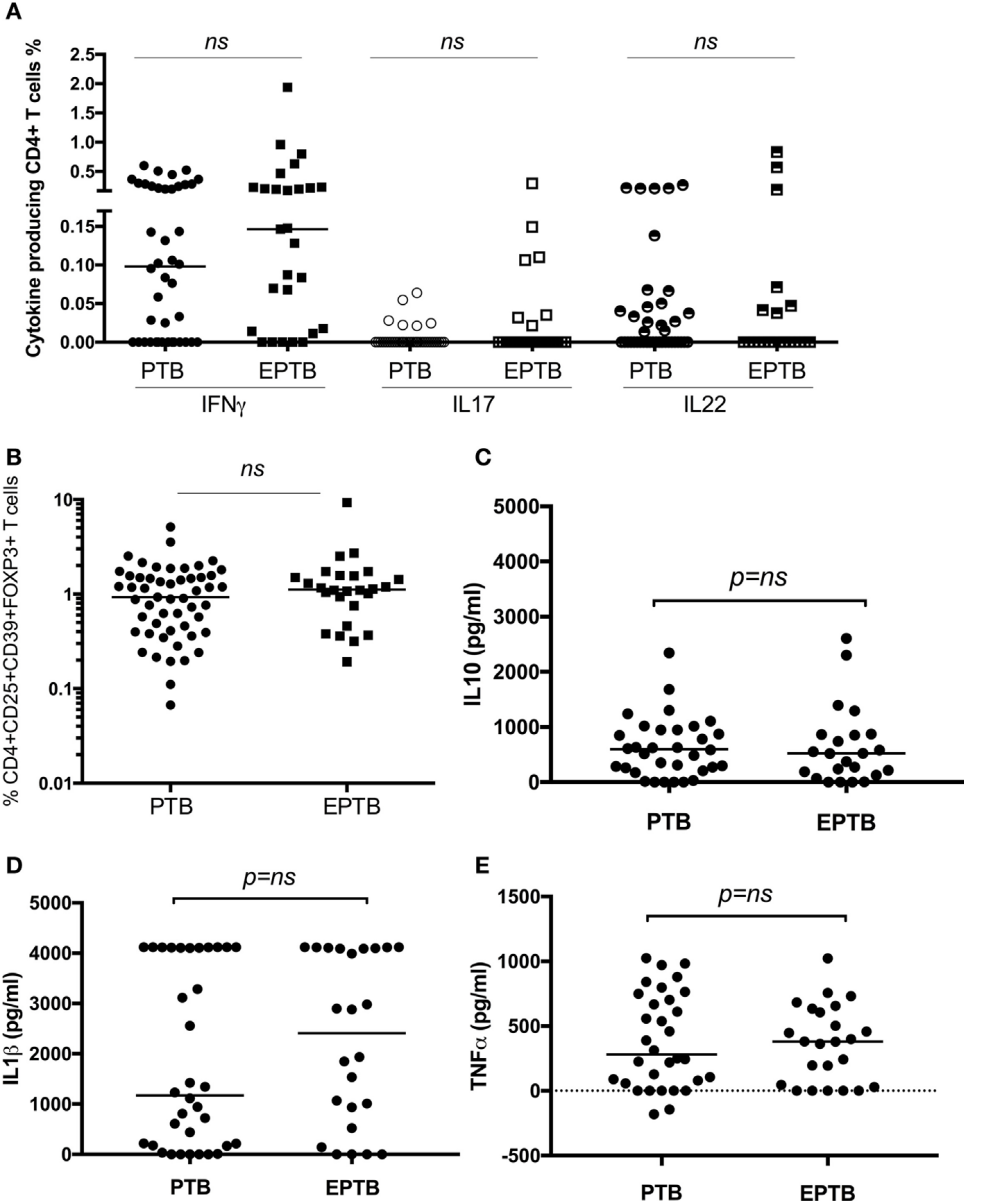
**T cell assays did not distinguish between children with pulmonary TB (PTB) disease and children with extrapulmonary TB (EPTB) disease**. Frequencies of IFNγ-, IL17-, and IL22-producing CD4^+^ T cells as detected by an intracellular cytokine assay following stimulation of whole blood with BCG for 16 h **(A)**, the frequency of CD4^+^CD25^+^CD39^+^FOXP3^+^ regulatory T cells in unstimulated whole blood **(B)**, and the secreted level of key cytokines following 6 days of stimulation with BCG **(C,D,E)** are shown. The assays were performed in both groups: PTB (*n* = 48) and EPTB (*n* = 28). Horizontal bars represent median values. The dotted line represents a cutoff of 0.01 for a positive response. *p*-Values calculated by Mann–Whitney are displayed.

As we have identified suppression of a number of pro-inflammatory and effector responses as described above in children with TB compared to healthy controls, we sought to establish whether these could distinguish between TB disease manifestations. We compared all immune parameters between children with PTB (*n* = 48) to EPTB (*n* = 28). This analysis did not reveal any significant differences in any of the T cell populations or their related cytokines between the groups (Figures 3A–E, not all data shown).

### Impact of TB Treatment on T Cell Responses

Our results show that children with disease have suppressed pro-inflammatory cytokine production and increased regulatory T cell frequency at the time of diagnosis. We wished to establish whether these were induced by TB disease or due to an underlying and persistent immune-phenotype, which might have pre-disposed this population to contract TB. We therefore, repeated all investigations in the children with TB disease following 6 months of TB treatment. Results post-TB therapy were compared to the results obtained at the time of diagnosis and to healthy controls at the time of enrollment. Follow-up time points were not included in healthy children as there was no intervention or prolonged follow-up required, clinically.

**Figure 4:**
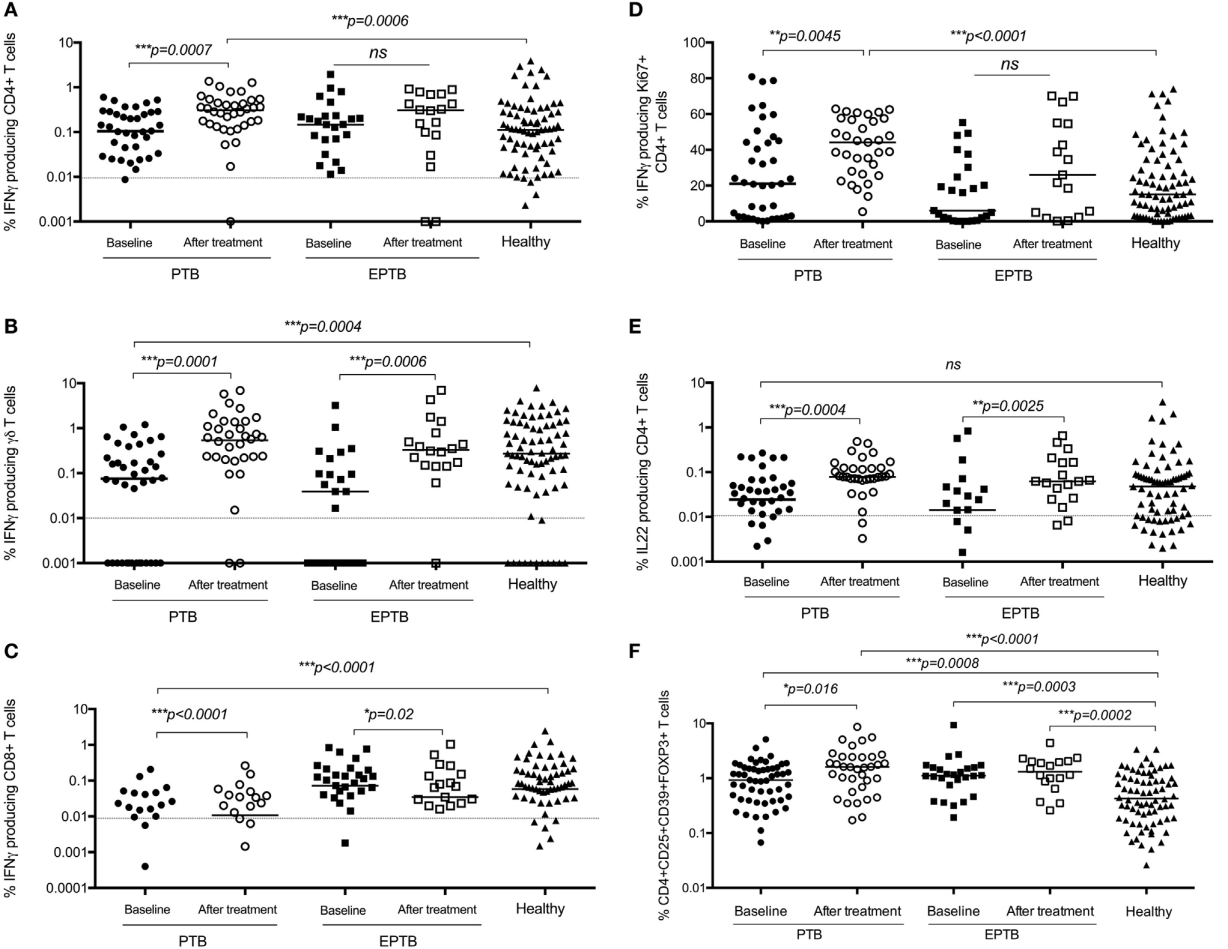
**BCG-induced IFNγ production by CD4^+^, CD8^+^, and γδ^+^ T cells and regulatory T cells are increased following treatment compared to diagnosis and compared to levels in healthy children**. Frequencies of IFNγ-producing CD4^+^ T cells **(A,D)**, γδ^+^ T cells **(B)**, or CD8^+^ T cells **(C)** or IL22-producing CD4^+^ T Cells **(E)** as detected by an intracellular cytokine assay following stimulation of whole blood with BCG for 20 h **(A,B,C,E)** or for 6 days **(D)** are shown. CD4^+^CD25^+^CD39^+^FOXP3^+^ regulatory T cells in unstimulated blood are shown **(F)**. The assays were performed in children with pulmonary TB (PTB) (*n* = 48), extrapulmonary TB (EPTB) (*n* = 28) at the time of diagnosis and following treatment for tuberculosis (TB), or at recruitment in the case of children with no evidence of TB sensitization or disease (healthy) (*n* = 83). Horizontal bars represent median values. The dotted line represents a cutoff of 0.01 for a positive response. *p*-Values calculated by Mann–Whitney are displayed.

Overall, almost all measured immune responses were significantly higher post treatment, and a number of significant differences between PTB and EPTB emerged. The frequency of BCG-specific CD4^+^ T cells producing IFNγ in both short term and proliferation assays was increased in children with PTB compared to the time of diagnosis and compared to healthy children (Figures 4A,D), but not in children with EPTB.

The frequency of regulatory T cells had also increased significantly in children with PTB by 6 months compared to the time of diagnosis, but not in those with ETPB (Figure 4F).

The frequency of BCG-specific γδ^+^ and CD8^+^ T cells producing IFNγ was increased in both assays in children with PTB and EPTB, but not in comparison to healthy children (Figures 4B,C).

There were no significant differences in production of IL17 in CD4^+^ or γδ^+^ T cells following treatment (data not shown). However, the production of IL22 by CD4^+^ T cells increased significantly following treatment in children with both pulmonary and extrapulmonary TB compared to baseline and healthy controls (Figure 4E).

## Discussion

Previous studies examining the immune response to M.tb in children have been limited in sample size and have primarily focused either on a single T cell phenotype or on secreted cytokines. Our study compared different clinical manifestations of TB disease and included a comprehensive range of T cell populations, using two different immunological assays to capture both effector and central memory T cell responses. Additionally, we tested these responses at time of diagnosis and following treatment.

We found an increased frequency of regulatory T cells in children with TB disease, not previously reported, although we did not see differences between children with PTB and EPTB. Our results, therefore, differ from adult data that reported a 2.3-fold increase in FOXP3 expression in patients with EPTB compared to PTB (12). The use of the surface marker CD39 may explain the difference between our findings and the limited studies in adults. It is also possible that regulatory T cells in young children behave differently from those in adult patients, where active TB is more likely to arise from reactivation or reinfection than the primary infection most commonly seen in young children. Infants have been shown to have greater numbers of CD45RA^+^ phenotypically naïve regulatory T cells which are functionally different, proliferating, and persisting for longer than the CD45RA-regulatory T cells which predominate in adults. The role of these different phenotypes of regulatory T cells may have been interesting to explore in this cohort (24).

Previous studies have demonstrated suppression of effector immune responses, such as IFNγ production by CD4 T cells in children with TB in comparison to healthy controls (25). Our data show lack of suppression of Th1 immune responses as measured by either the IFNγ production by CD4^+^ T cells or the Ki67 proliferative capacity of these T cells. However, we found suppression of Th17 responses (IL17 and IL22 production by CD4 and γδ T cells) as well as significantly lower levels of secreted pro-inflammatory cytokines including IL1β, TNFα, and IFNγ, following stimulation of whole blood with BCG for 6 days. This finding is in line with adult data, which also demonstrated suppressed levels of antigen-specific immune responses to mycobacteria at the time of diagnosis (26, 27) and more recently a small pediatric study reporting suppressed IFNγ effector responses, which were measured as secreted cytokines following PPD stimulation after 24 h (28). A number of hypotheses have been formulated regarding this phenomenon of antigen-specific suppression of immune responses, including compartmentalization of relevant cell populations at the site of disease or an active and selective depletion of mycobacteria-specific T cells during TB disease (11, 26, 29). It is also possible that M.tb itself induces relative immunosuppression as a mechanism for survival in the host, in line with transcriptomic data which show downregulation of immune response genes in the presence of M.tb in both adults and children (17–20). Whether these findings represent “the chicken or the egg” remains to be fully established, but the fact that T cell responses recover following treatment in the PTB cohort might indicate that it is induced by the disease rather than representing a persistent underlying host immuno-phenotype.

In addition to regulatory T cells and CD4 T cell responses, our study also characterized the role of γδ T cells in the immune response to mycobacteria in children with TB. We report that although γδ T cells expand, proliferate, and produce both Th1 and Th17 cytokines in response to BCG stimulation in children with different manifestations of TB disease, they do not appear to behave in a counter-regulatory fashion to CD4 T cells, as we had originally hypothesized. Dieli et al. showed increased proliferative capacity of γδ T cells, but decreased IFNγ production in children with TB compared to healthy controls and an increase in IFNγ production after treatment (30). Similarly, our data demonstrate that BCG-induced γδ T cells produce significantly less IFNγ and IL17 in children with active TB disease at the time of diagnosis compared to healthy controls, but increase following treatment. Interestingly, we did not demonstrate a correlating suppression of IL22 production in active TB at the time of diagnosis compared to healthy children. In non-human primates with severe TB disease, unbalanced upregulation of immune gene networks, including overexpression of IL22, was demonstrated by Yao et al. (31). Scriba et al. described a distinct subset of IL22-producing T cells in the lungs of adult patients with TB disease, and IL22 was found to be more abundant in both blood and at the disease site of patients with pericardial TB disease (29, 32). γδ T cells have previously been identified as the source of IL22 in adults with TB disease (33). IL22 is known to either enhance a pro-inflammatory state in the presence of IFNγ, TNFα, IL2, and IL17 or in their absence is recognized to have immune regulatory and regenerative functions. In our cohort, this latter immune regulatory state appears to predominate, with increased regulatory T cells and decreased IFNγ and TNFα present in children with active TB disease.

Despite measuring immediate and proliferative responses, the assays performed in this study were unable to distinguish between pulmonary and extrapulmonary TB. A number of studies have explored this previously with discordant results. Verbon et al. demonstrated that unstimulated cytokine levels in serum were present in equivalent levels in pulmonary and extrapulmonary TB, while Sharma et al. recorded higher levels in miliary TB, a form of severe extrapulmonary TB, compared to pleural TB (34, 35). Conversely, a number of studies have demonstrated that both unstimulated and stimulated levels of pro-inflammatory cytokines are present in lower levels in adults with extrapulmonary or severe TB disease compared to pulmonary TB (36, 37), both at the time of treatment and afterward (38), leading to suggestions of an underlying immune defect in those patients who develop extrapulmonary TB disease. In particular, regulatory T cells and circulating levels of IL10 have been suggested to be increased in patients with extrapulmonary TB both during the disease period and subsequently (13, 36, 39). These studies were performed in adults who are likely to present with reactivation disease and high bacterial loads, unlike children who usually develop primary progressive paucibacillary disease. It is, therefore, not surprising that these results differ from our own findings and between study populations.

We repeated all assays in our TB cohorts following completion of 6 months treatment to determine whether the study findings were possibly due to an underlying susceptibility for TB disease or represented a phenomenon induced by active disease. In agreement with other investigators, our data show recovery of the antigen-specific responses post treatment to levels that were of greater magnitude than those reported in healthy children. This would indicate that their suppression in face of acute disease represents a pathogen-induced response. Interestingly, the recovery response was of lesser magnitude in children with EPTB. Whether this may be attributable to higher bacillary burden at the onset of disease or slower clearance rates of mycobacteria cannot be determined, as we did not have the capacity to collect this information as part of our study. It is, however, recognized that patients with ETPB have higher rates of culture confirmation and relapse than those with PTB, justifying the usually longer treatment course. Another explanation may be that these children represent a cohort in whom the original BCG vaccine was less effective, but given that there were no significant differences in acquired immune responses as measured by BCG-specific, IFNγ-producing CD4 T cells at the time of diagnosis, this seems unlikely. Published data have already shown that IFNγ responses measured at 10 weeks post BCG vaccination in a Cape Town Cohort could not distinguish between infants at risk of TB disease over time, further refuting this possibility (5).

Our data show that the frequency of regulatory T cells is increased in both PTB and EPTB following treatment. While this could be explained at the time of diagnosis as a response to the presence of high levels of antigen, it is an unlikely explanation after 6 months of effective treatment for TB. This may represent an underlying susceptibility to TB disease in these children. Alternatively, the memory recall response to BCG, which results in expansion of effector T cells at the end of treatment, may equally induce the expansion of regulatory T cells.

A unique strength of our study is the number of children recruited and their thorough clinical and immunological characterization within distinct groups, including healthy controls. It is noteworthy that there were no significant differences between the immune responses to mycobacteria in the clinically diagnosed vs microbiologically confirmed TB groups. It was also somewhat surprising that the median age of children with EPTB was 5 years, since EPTB is historically associated with younger children. The median age of 96 children with EPTB recruited as part of a parallel diagnostic study in Cape Town was 48.9 months also (personal communication). This may reflect protection conferred by BCG vaccination at birth or could represent a location bias because of recruitment in hospital rather than in the community. Additionally, much of the EPTB was either bone, joint, or pleural disease, rather than miliary or disseminated disease which is normally described in the younger infants.

A limitation of our study is the relatively short duration of follow-up of the TB cohort for 6 months. Repeating the assays after a period of 2 years to determine whether the increased responses observed following 6 months of treatment returned to healthy control levels with time could be of interest. Despite designing our study to be age-matched with the expectation that children under 2 years would present with extrapulmonary TB, the children with extrapulmonary TB were older. Although some immune responses are known to mature with age, antigen-specific immune responses to BCG in infants are comparable to those seen in adults (40). A further limitation of the study was the heterogeneity of disease in the EPTB cohort, as children with bone or joint TB may represent a different immunological pathogenesis than disseminated miliary TB or TB meningitis. The median age remained the same in the subgroups, but the numbers in each group were too small to reach significant conclusions. A further limiting factor is that the assays were performed only in blood, which may not reflect mechanisms of disease in the lung or other sites of disease, but obtaining samples from the site of disease in children is challenging.

In conclusion, we report for the first time that regulatory T cells are increased in patients with active TB disease, but that they do not distinguish between EPTB and PTB. CD4 effector T cell responses, typically used in diagnostics and as markers of vaccine efficacy, do not distinguish between TB disease and healthy children in this cohort, but we report suppressed levels of pro-inflammatory cytokines and γδ T cells cytokine responses in children with TB disease compared to healthy controls. However, this does not explain why some children develop EPTB and others PTB. Further investigation of the relationship between regulatory T cells and innate immune responses is required to define correlates of immune protection associated with clinical manifestations of childhood TB.

## Ethics Statement

This study was carried out in accordance with the recommendations of Research Ethics Committee of the Faculty of Health Sciences, University of Cape Town with written informed consent from all subjects. All subjects gave written informed consent in accordance with the Declaration of Helsinki. The protocol was approved by the Research Ethics Committee of the Faculty of Health Sciences, University of Cape Town.

## Author Contributions

EW and BK designed the study and experiments, performed the data interpretation. EW performed the experiments, data capture, and analysis. All authors contributed to the writing and/or review of the manuscript.

## Conflict of Interest Statement

The authors declare that the research was conducted in the absence of any commercial or financial relationships that could be construed as a potential conflict of interest. The reviewer, MO, declared a shared affiliation, though no other collaboration, with the authors, EW and BK, to the handling editor, who ensured that the process nevertheless met the standards of a fair and objective review.
